# Acute complications of giant coronary aneurysms in Kawasaki disease

**DOI:** 10.1093/ehjcr/ytae586

**Published:** 2024-10-29

**Authors:** Lucia D Beissel, Christopher Hart, Julian A Luetkens

**Affiliations:** Department of Diagnostic and Interventional Radiology, University Hospital Bonn, Venusberg-Campus 1, Bonn 53127, Germany; Quantitative Imaging Laboratory Bonn (QILaB), University Hospital Bonn, Venusberg-Campus 1, Bonn 53127, Germany; Department of Diagnostic and Interventional Radiology, University Hospital Bonn, Venusberg-Campus 1, Bonn 53127, Germany; Department of Pediatric Cardiology, University Hospital Bonn, Venusberg-Campus 1, Bonn 53127, Germany; Department of Diagnostic and Interventional Radiology, University Hospital Bonn, Venusberg-Campus 1, Bonn 53127, Germany; Quantitative Imaging Laboratory Bonn (QILaB), University Hospital Bonn, Venusberg-Campus 1, Bonn 53127, Germany

**Figure:**
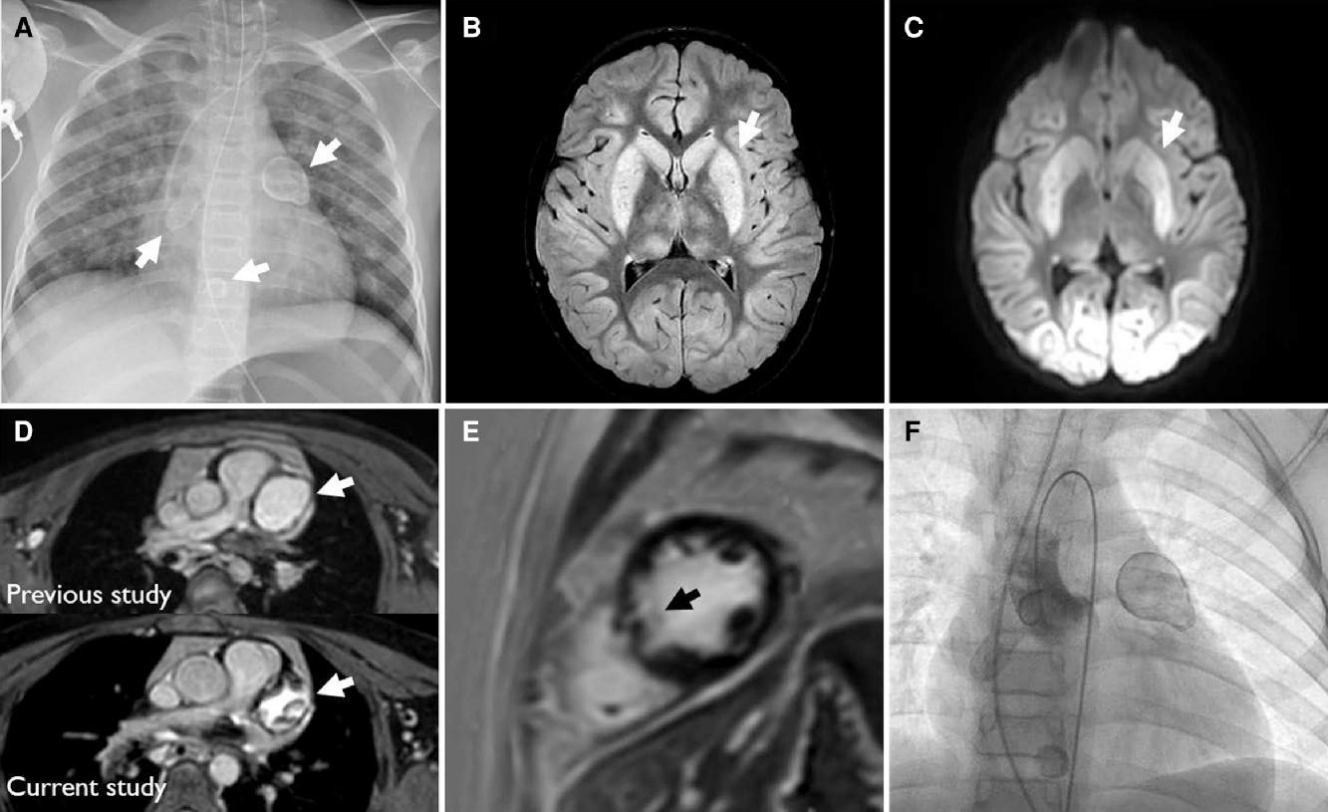


An 8-year-old female patient with Kawasaki disease presented to the emergency department with return of spontaneous circulation after 40 min of resuscitation due to ventricular fibrillation. Chest X-ray showed three giant coronary aneurysms with calcified margins in the proximal left anterior descending (LAD) and proximal and distal right coronary artery (RCA) (*Panel A*, arrows). The coronary aneurysms were already known before and were so far treated with immunoglobulin, aspirin, cortisone, and infliximab.

Magnetic resonance imaging of the brain revealed hypoxic brain injury involving the basal ganglia and the central region on ‘fluid-attenuated inversion recovery’ and diffusion-weighted sequences (*Panels B* and *C*, arrows).

The patient regularly receives cardiovascular magnetic resonance (CMR) check-ups since her diagnosis of Kawasaki at the age of 3 months. A renewed CMR revealed new partial thrombosis of the proximal coronary aneurysms and marginal calcification of the distal RCA aneurysm compared to a check-up examination 2 years ago (*Panel D*, arrows). Late gadolinium enhancement showed a new septal perforator branch infarction—primarily triggered by coronary artery occlusion because of the thrombosis—as the cause of the initial resuscitation (*Panel E*, arrow). Cardiac catheterization showed reduced contrast media flow in the RCA and LAD (*Panel F*, Supplementary material online, *Video S1*). For secondary prophylaxis, an implantable cardioverter defibrillator was implanted. Left and right ventricular ejection fraction were normal on follow-up (see Supplementary material online, *Video S2*). However, the patient shows persistent neurological abnormalities due to the hypoxic brain damage. She is awake, but only responds to speech with sounds and grimacing and exhibits spasticity of the lower extremities and swallowing disorders.

Kawasaki disease—a vasculitis of the small- and medium-sized arteries—mostly affects children under the age of 5 years. A fever lasting at least 5 days and at least four main symptoms allow the diagnosis. Main symptoms are exanthema, conjunctivitis, stomatitis, involvement of the extremities, and cervical lymphadenopathy. Diagnosis can be difficult in infants under the age of 6 months who often only present misinterpretable fever and irritability. Though, this patient group has a high risk to develop coronary aneurysms. Therefore, incomplete Kawasaki disease should be considered in the presence of prolonged unexplained fever, less than four of the five major symptoms, and consistent laboratory and echocardiographic results.

This case should sensitize to early diagnose and initiate therapy in Kawasaki patients that can drastically reduce the risk of coronary aneurysms and possible development of complications.

## Supplementary Material

ytae586_Supplementary_Data

## Data Availability

No new data were generated or analysed in support of this research.

## Notes

**Consent:** The authors confirm that written consent for the submission and publication of this case report, including images and associated text, was obtained from the patient’s legally authorized representatives (parents), in line with the COPE guidelines.

**Funding:** None declared.

